# Dysregulation of Cell Survival in Diffuse Large B Cell Lymphoma: Mechanisms and Therapeutic Targets

**DOI:** 10.3389/fonc.2019.00107

**Published:** 2019-03-01

**Authors:** Yi Miao, L. Jeffrey Medeiros, Zijun Y. Xu-Monette, Jianyong Li, Ken H. Young

**Affiliations:** ^1^Department of Hematopathology, The University of Texas MD Anderson Cancer Center, Houston, TX, United States; ^2^Department of Hematology, The First Affiliated Hospital of Nanjing Medical University, Jiangsu Province Hospital, Nanjing, China; ^3^Graduate School of Biomedical Sciences, The University of Texas Health Science Center at Houston, Houston, TX, United States

**Keywords:** DLBCL, cell survival, apoptosis, BCR signaling, BCL2, p53, TME, EBV

## Abstract

Diffuse large B cell lymphoma (DLBCL) is the most common type of lymphoma worldwide, representing 30–40% of non-Hodgkin lymphomas, and is clinically aggressive. Although more than half of patients with DLBCL are cured by using standard first-line immunochemotherapy, the remaining patients are refractory to the first-line therapy or relapse after complete remission and these patients require novel therapeutic approaches. Understanding the pathogenesis of DLBCL is essential for identifying therapeutic targets to tackle this disease. Cell survival dysregulation, a hallmark of cancer, is a characteristic feature of DLBCL. Intrinsic signaling aberrations, tumor microenvironment dysfunction, and viral factors can all contribute to the cell survival dysregulation in DLBCL. In recent years, several novel drugs that target abnormal cell survival pathways, have been developed and tested in clinical trials of patients with DLBCL. In this review, we discuss cell survival dysregulation, the underlying mechanisms, and how to target abnormal cell survival therapeutically in DLBCL patients.

## Introduction

Diffuse large B cell lymphoma (DLBCL) is the most common type of lymphoma and represents 30–40% of all non-Hodgkin lymphomas (1). Based on gene expression profiling, two molecular subtypes of DLBCL, activated B-cell-like (ABC) subtype and germinal center B-cell-like (GCB) subtype, are identified (2, 3). Approximately 60% of patients with DLBCL can be cured by the standard immunochemotherapy regimen rituximab plus cyclophosphamide, doxorubicin, vincristine, and prednisone (R-CHOP), but remaining patients are either refractory to induction immunochemotherapy or experience relapses after achieving complete response (CR) (4–6). Enormous effort has been devoted to developing new therapeutic approaches for these refractory or relapsed patients, with some success. In rituximab era, using conventional salvage immunochemotherapy with autologous transplant, only ~ 10% of refractory/relapsed DLBCL cases could be cured, whereas the remaining patients have very dismal outcome warranting development of novel therapies (7, 8). Understanding the pathogenesis of DLBCL is vital to define potential therapeutic targets and develop regimens for the treatment of DLBCL (9, 10). As is common to other types of cancer, dysregulation of cell survival or resistance to cell death also contributes to the pathogenesis of DLBCL. DLBCL lymphoma cells have evolved many strategies to resist cell death, which potentially can be therapeutically targeted. In this review, we discuss the mechanisms underlying dysregulation of cell survival in DLBCL and therapeutic options to target related pathways.

## Mechanisms Underlying Dysregulation of Cell Survival in Dlbcl

### B Cell Receptor (BCR) Signaling

#### Normal BCR Signaling

**Figure 1:**
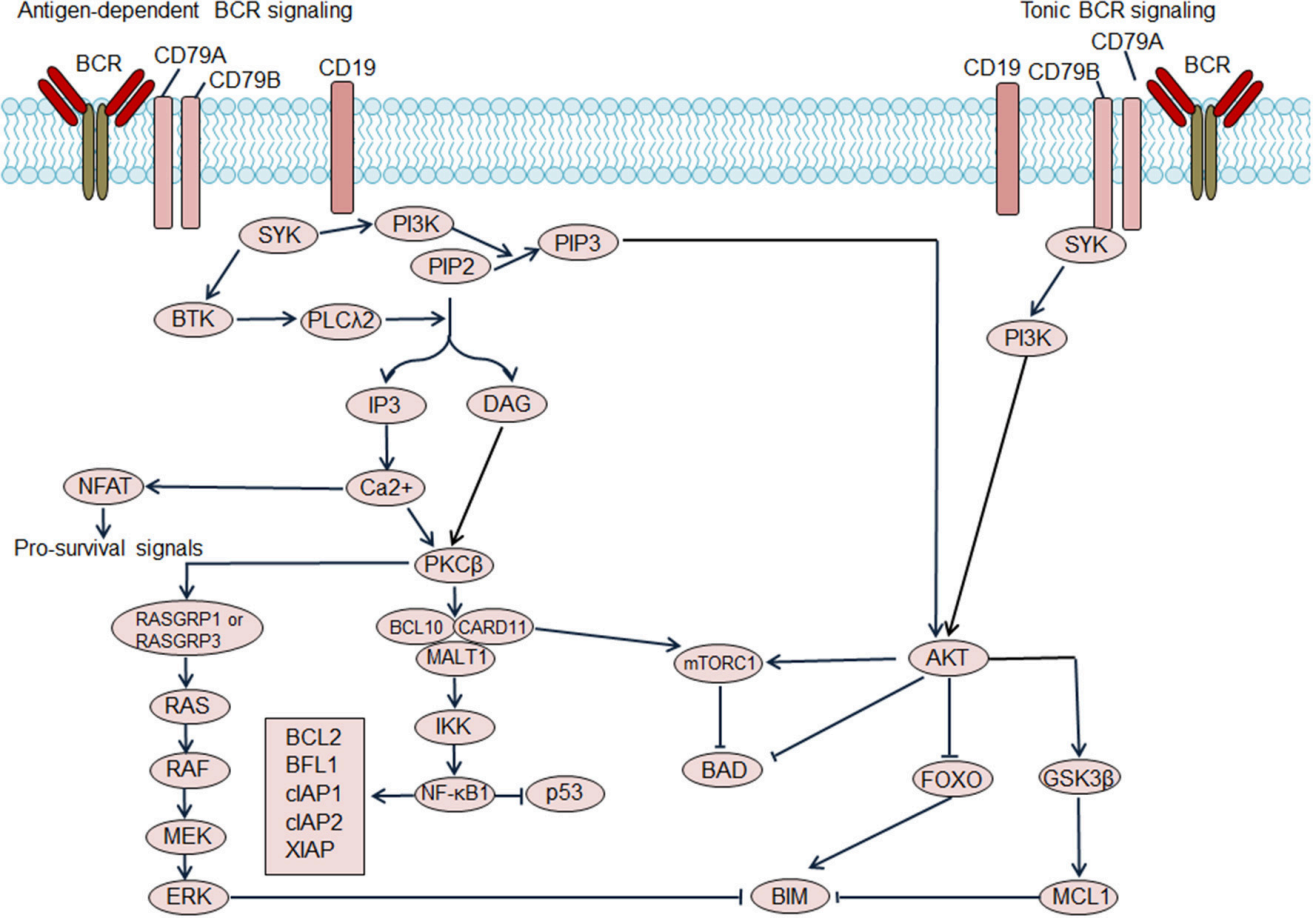
Regulation of B cell receptor (BCR) signaling on B cell survival. Antigen dependent BCR signaling engages several downstream pathways, which include the NF-κB pathway, PI3K/AKT/mTOR pathway, NFAT pathway, and MAPK/ERK pathway. NF-κB activation promotes transcription of the pro-survival genes including BCL2, BFL1, cellular inhibitor of apoptosis protein (cIAP) 1, cIAP2, X-linked inhibitor of apoptosis protein (XIAP) and so on. Additionally, NF-κB antagonizes the function of p53, thereby decreasing p53-mediated cell apoptosis. Activated AKT phosphorylates forkhead box class O family member transcription factors (FOXOs), suppressing the transcription of target genes including pro-apoptosis gene BIM. AKT activation also results in the activation of pro-survival MCL1. Moreover, AKT mediates the phosphorylation of the pro-apoptotic BCL2 family member BAD, and blocks BAD-induced cell apoptosis. The activation of mTOR complex 1 (mTORC1), which suppresses BAD, could be induced by AKT activation or CARD11 activation. Tonic BCR signaling triggers activation of the PI3K/AKT pathway and also promotes cell survival. BTK, Bruton tyrosine kinase; DAG, diacylglycerol; IP3, inositol trisphosphate; PIP2, phosphatidylinositol-4,5-bisphosphate; PIP3, phosphatidylinositol (3,4,5)-trisphosphate; PKCβ, protein kinase C β; PLCλ2, phospholipase λ2; SYK, spleen tyrosine kinase.

Based on dependence on antigen stimulation, BCR signaling pathways can be categorized into tonic BCR signaling or antigen-dependent BCR signaling. Tonic BCR signaling is vital for maintaining the survival of resting mature B cells, as ablation of cell surface BCR expression or inactivation of BCR result in cell death (Figure 1) (11). Tonic BCR signaling exerts its pro-survival effects via PI3K signaling, as PI3K signaling can rescue the survival of mature B cells that are deficient in BCR (12). Activated PI3K/AKT inactivates the pro-apoptotic protein BAD and stabilizes the anti-apoptotic protein MCL1, resulting in enhanced cell survival. Moreover, activated AKT phosphorylates forkhead box class O family member transcription factors (FOXOs) leading to cytosolic retention of FOXOs, impairing FOXOs-mediated transactivation of cell death genes (12). Antigen-dependent BCR signaling promotes the survival of B cells through activation of a variety of pathways. The binding of antigen causes BCR aggregation, leading to phosphorylation of immunoreceptor tyrosine-based activation motifs (ITAMs) of CD79A and CD79B by SRC kinases, including LYN, FYN, and B lymphocyte kinase (BLK). Phosphorylated ITAMs recruit spleen tyrosine kinase (SYK), which is then phosphorylated and activated by the SRC kinases and autophosphorylation. Activated SYK leads to phosphorylation and activation of the downstream molecules Bruton tyrosine kinase (BTK) and phospholipase Cγ2 (PLCγ2) (9). PLCγ2 hydrolyses phosphatidylinositol-4,5-bisphosphate (PIP2) into diacylglycerol (DAG) and inositol trisphosphate (IP3), which results in increased intracellular calcium levels. Increased calcium, in combination with DAG, triggers activation of protein kinase C β (PKCβ) and promotes assembly of the CARD11/BCL10/MALT1 (CBM) complex and NF-κB activation, providing vital pro-survival signals for B cells. In BCR signaling, LYN kinase also leads to the phosphorylation of CD19 thereby promoting recruitment recruitment of PI3K to the BCR, which then activates the PI3K/AKT pathway. Additionally, BCR signaling can also activate the RAS and mitogen-activated protein kinase (MAPK) pathway as well as the transcription factor NFAT, providing additional survival signals for B cells (9).

#### Chronic Active BCR Signaling

ABC DLBCL cells rely on BCR signaling for survival (13). This type of BCR signaling is dependent on both proximal BCR pathway components and CARD11, similar to that in antigen activated normal B cells, therefore referred as “chronic active” BCR signaling (13). Down-regulation of chronic active BCR signaling in ABC DLBCL cells leads to inhibition of NF-κB activation as well as phosphorylation of AKT and ERK, dampening pro-survival signaling (13). Both antigen-dependent BCR activation and genetic aberrations involving the BCR signaling pathway contribute to chronic active BCR signaling activation in ABC DLBCL (13). One part of the evidence suggesting antigen-dependent BCR activation in ABC DLBCL is remarkable clustering of BCR on the ABC DLBCL cell surface, a characteristic feature of antigen dependent BCR activation in normal B cells (13). A study by Young et al. showed, in a subset of ABC DLBCL, that binding of self-antigen to BCR drove active BCR signaling and sustained survival of ABC DLBCL cells (14). Genetic aberrations affecting BCR and downstream signaling contribute to the activation of BCR signaling in ABC DLBCL. A subset of ABC DLBCL cases harbor mutations in the ITAM motif of CD79A (~3%) and CD79B (~20%) (13). Both CD79A and CD79B mutations enhance surface IgM expression, thereby increasing NF-κB activation. *CD79B* mutations also suppress the activity of LYN kinase, impairing LYN kinase mediated negative-feedback inhibition of BCR signaling. Consistently, inactivating *LYN* mutations, and *LYN* deletions have been identified in DLBCL, which possibly enhance activation of BCR signaling. The CARD11/BCL10/MALT1 complex is also affected by activating mutations or amplifications. *CARD11* mutations, which predominantly affect the coil-coil domain, are detected in DLBCL (11–15%) including both ABC and GCB DLBCL (15, 16). These *CARD11* mutations impair the inhibition of domain-mediated auto-inhibition, leading to hyper-activation of CARD11, which subsequently activates the downstream NF-κB pathway (17). A recent study showed that activated CARD11 could induce the activation of mTOR complex 1 (mTORC1), which provides additional pro-survival signals (18). With the advent of next-generation sequencing, an increasing number genetic aberrations of BCR regulators have been identified, especially negative BCR regulators, including PTPN6, PRKCD, SLA, LAPTM5, DGKZ, and MAP4K1 (16). The inactivating mutations or deletions involving these molecules release BCR signaling from inhibition, thus leading to BCR signaling activation.

#### Tonic BCR Signaling

Absence of immobile BCR clustering on the cell surface of GCB DLBCL cells suggests lack of chronic active BCR signaling. Moreover, most GCB DLBCLs are relatively insensitive to the BCR inhibitor ibrutinib and do not show activation of NF-κB pathway, further suggesting independence of GCB DLBCL from chronic active BCR signaling (19). The study by Chen et al. suggested some DLBCL cell lines, which included GCB subtypes, displayed tonic BCR signaling, as these cell lines exhibited detectable SYK and BLNK phosphorylation without BCR crosslinking (20). Inhibition of SYK dampened tonic BCR signaling and increased cell apoptosis in BCR-dependent DLBCL cell lines, pointing to a role of tonic BCR signaling in sustaining survival of BCR-dependent DLBCL cells (20). Replacement of BCR antigen-binding regions has no impact on BCR signaling in GCB DLBCL lines, indicating that GCB DLBCL rely on tonic BCR signaling (21). The biological effect of tonic BCR signaling in GCB DLBCL is highly dependent on AKT activation, as tonic BCR signaling triggers AKT activation and forced AKT activation can rescue GCB DLBCL cells from depletion of the BCR or tonic BCR signaling mediators SYK and CD19 (21). Genetic aberrations also play a role in promoting tonic BCR signaling. *PTEN* deletions, which are identified in approximately 10% of DLBCL including the GCB and ABC subtypes, can result in enhanced PI3K/AKT signaling (16). Mir-17-92 targets and negatively regulates expression of PTEN protein, therefore, mir-17-92 amplification, which occurs exclusively in GCB DLBCL (~8%) (16), leads to PI3K/AKT activation. These aberrations, by activating PI3K/AKT signaling, lead to increased tonic BCR signaling.

### Toll-Like Receptor Signaling and the MyD88–TLR9–BCR Supercomplex

*MYD88*^L265P^ mutation, which involves the adaptor protein MYD88 in the Toll-like receptor (TLR) pathway, is found in approximately 30% of ABC DLBCL, suggesting that abnormal TLR signaling plays a role in the pathogenesis of DLBCL (22). Physiologically, following recruitment of MYD88 to TLRs, TLR4 forms a complex with interleukin-1 receptor-associated kinase (IRAK) 4, further recruiting other IRAKs (IRAK1 and IRAK2) and causing activation of downstream pathways including the NF-κB pathway (23). Inhibiting MYD88 using shRNA is toxic to ABC DLBCL cell lines suggesting that MYD88 is essential for the survival of these cell lines (24). Compared with wild-type MYD88, MYD88^L265P^ leads to enhanced phosphorylation of IRAK, activation of NF-κB, and JAK-STAT3 signaling, and increased secretion of interleukin 6 (IL-6), interleukin 10 (IL-10), and interferon-β promoting the survival of ABC DLBCL cell lines. The study by Avbelj et al. found that MYD88^L265P^ Toll/interleukin-1 receptor (TIR) domain can recruit endogenous wild-type MYD88 to form oligomers, which leads to hyperactivation of downstream pathway (25). Moreover, conditional expression of *MYD88*^L265P^ mutation in B cells in mice leads to spontaneous development of lymphoproliferative diseases which mimic ABC DLBCL (26). These data suggest that MYD88^L265P^ activates MYD88, promotes the survival of DLBCL cells, and contributes to the pathogenesis of ABC DLBCL. Additional conditional BCL2 overexpression in this model remarkably accelerates the development of lymphoma (26). Accordingly, in human DLBCL samples, *BCL2* amplification frequently co-occur with *MYD88*^L265P^ mutations in ABC DLBCL, suggesting the anti-apoptotic function of BCL2 is important for full transformation of B cells by MYD88^L265P^ (27). This finding also provides a rationale for using BCL2 inhibitors in *MYD88*^L265P^ mutated DLBCL.

Additionally, Co-occurrence of *MYD88*^L265P^ and *CD79B* mutations is frequent in ABC DLBCL, suggesting that these two aberrations might be synergistic in driving ABC DLBCL development (27). There has been direct evidence that MYD88 and BCR cooperate in the pathogenesis of a subset of DLBCL (28). A recent study showed that MYD88, TLR9, and the BCR formed a multiprotein supercomplex (MyD88–TLR9–BCR supercomplex, the My-T-BCR supercomplex) in ibrutinib-responsive cell lines and patient samples (28). The My-T-BCR supercomplex co-localizes with mTOR on endolysosomes to drive NF-κB and mTOR signaling, both of which promote cell survival (28).

### Dysregulation of Apoptosis Molecules

#### Dysregulation of BCL2 Family Members

The BCL2 family consists of a group of proteins that share with Bcl-2 homology (BH) domains (29). BCL2 family proteins, including anti-apoptotic and pro-apoptotic members, have a crucial role in regulating cell survival by modulating the intrinsic apoptosis pathway. Briefly, signaling including DNA damage and absence of growth factors leads to the activation of BH3-only proteins, which inactivate the pro-survival members such as BCL2, allowing activation of BAX and BAK. BAX and BAK lead to permeabilization of the outer mitochondrial membrane, releasing the pro-apoptotic cytochrome c, which activates caspases. These caspases, via their proteolytic activities, act as the direct mediators of cell apoptosis.

Dysregulation of BCL2 family members has been reported in DLBCL. BCL2, the prototype of this family, is overexpressed in 50%-53% of DLBCL (ABC DLBCL, 53–61%; GCB DLBCL, 40–44%) (30, 31). BCL2 contributes to the pathogenesis of DLBCL by promoting the survival of B cells, as *BCL2*-transgenic mice have B cells with extended survival and these mice spontaneously develop aggressive B cell lymphoma (32–34). Several mechanisms contribute to BCL2 overexpression in DLBCL, among which *BCL2* translocation is the most common one. *BCL2* translocation occurs exclusively in GCB DLBCL (~30%) (16, 35, 36), which juxtaposes *BCL2* with the immunoglobulin heavy chain (IGH) enhancer, leading to increased *BCL2* mRNA transcription. *BCL2* copy number alterations, including gains and amplifications, are observed in ABC DLBCL and regarded as contributing factors to BCL2 overexpression (37). In normal germinal center (GC) B cells, BCL6 binds to the promoter of *BCL2*, and inhibits MIZ1-mediated *BCL2* transcriptional activation (38). *BCL2* translocations as well as mutations in *BCL2* promoter regions, disrupt BCL6-mediated suppression of *BCL2* transcription, thereby contributing to BCL2 overexpression (38). MicroRNA (miRNA) dysregulation also contributes to BCL2 overexpression in DLBCL (39). For instance, miR-34a, which targets BCL2, is down-regulated in DLBCL, contributing to BCL2 overexpression (39). Mutations in *BCL2* coding sequence (CDS), which is located predominantly in the BH4 and flexible loop domain (FLD), also contribute to the BCL2 dysregulation by affecting the function and stability of BCL2 (40–42). The anti-apoptotic BCL2 family member MCL1 is also overexpressed in non-GCB DLBCL (~50% at protein level), which could be caused by *MCL1* gains or amplification or abnormal activated STAT3 signaling (43). MCL1 possibly contributes to the pathogenesis of DLBCL by promoting cell survival, as *MCL1* transgenic mice spontaneously develop DLBCL and inhibition of MCL1 in DLBCL cell lines induces cell death (43, 44). Another BCL2 family anti-apoptotic member, BCL-w, has been shown to be overexpressed in ~90% of DLBCL (60% with high expression) and BCL-w overexpression was associated with poor prognosis (45, 46). The RNA level of BCL-w is inversely correlated with that of BCL2 in DLBCL samples, pointing to a possible complementary role of BCL-w to BCL2 in the pathogenesis of DLBCL (46). Loss of BCL-w sensitized B cell to growth factor deprivation-induced cell apoptosis and suppressed MYC-induced lymphomagenesis, suggesting a crucial role of BCL-w in B cell survival and lymphoma development (45). Nevertheless, the mechanisms underlying BCL-w overexpression in DLBCL remain not well-defined. In summary, abnormalities in anti-apoptotic members of the BCL2 family, such as BCL2, MCL1, and BCL-w, lead to enhanced B cell survival and contribute to DLBCL development.

#### Dysregulation of Inhibitor of Apoptosis Proteins

Inhibitor of apoptosis proteins (IAPs) inhibit cell apoptosis by binding specific caspases. Dysregulation of IAPs has been reported in DLBCL. Cellular inhibitor of apoptosis protein 1 (cIAP1), cellular inhibitor of apoptosis protein 2 (cIAP2), and X-linked inhibitor of apoptosis protein (XIAP) are reported in 92, 37, and 26~55% of DLBCL, respectively (47, 48). And the overexpression of XIAP in DLBCL is significantly associated with worse outcome (48). Pharmacological inhibition of XIAP induces cell apoptosis in DLBCL cell lines, supporting the role of XIAP in maintaining cell survival of DLBCL cells (48). Survivin, another member of the inhibitor of apoptosis protein family, has been found to be overexpressed in ~60% of DLBCL (49). And in ABC DLBCL, survivin overexpression significantly predicted worse outcome (49). These pieces of evidence suggest that survivin has a pathogenic role in DLBCL.

#### TP53 Dysregulation

**Figure 2:**
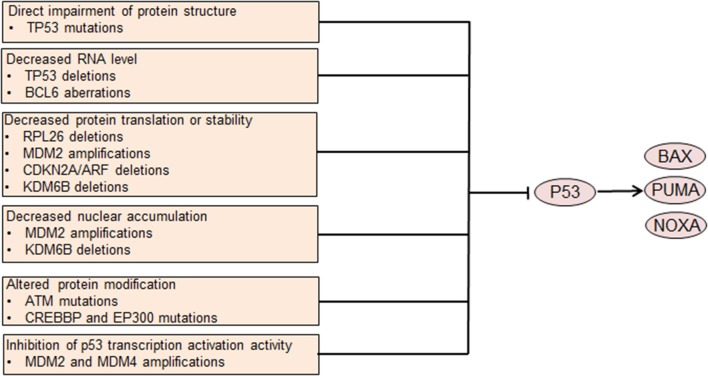
Genetic aberrations causing p53 dysfunction leads to dysregulated cell survival. Mutations involving the coding DNA sequence region of the *TP53* gene affect the protein structure and abrogate p53 tumor suppressor function. Other aberrations lead to decreased p53 translation or stability or altered protein modification. MDM2 and MDM4 also inhibit p53-mediated transcription activation. Decreased p53 nuclear accumulation caused by MDM2 amplifications or KDM6B deletions leads to p53 dysfunction. Impaired p53 function leads to decreased transcription of pro-apoptotic genes, resulting in abnormally enhanced cell survival.

The p53 transcription factor has a crucial role in regulating cell survival. Under normal conditions, the E3 ligase MDM2 targets p53 for degradation in a negative feedback manner, thus maintaining a low expression level of p53. Additionally, MDM2 suppresses p53 mediated transcriptional activation and promotes the transportation from the nucleus to the cytoplasm (50, 51). Cellular stresses, including DNA damage, can disrupt the binding of MDM2 to p53 and increase p53 protein expression. P53 activates transcription of several genes that are involved in cell apoptosis, including BAX, PUMA, and NOXA (Figure 2). TP53 dysfunction is common in DLBCL (52) and restoration of p53 expression tumor cell induces apoptosis in an Eμ-*Myc* mouse model, suggesting a role of p53 dysregulation in DLBCL development (53). P53 dysregulation also contributes to chemoresistance in DLBCL, as p53 functions as an important mediator of chemotherapy induced cell death. Clinically, *TP53* mutation is an independent prognostic factor of poor outcome in DLBCL patients treated with R-CHOP chemotherapy (54).

Many different mechanisms contribute to p53 dysregulation in DLBCL. Among these mechanisms, *TP53* mutation is the most common one, occurring in ~20% of all DLBCL cases (16, 52). *TP53* mutations in the CDS regions mostly target exons 4 through 9, frequently involving the DNA binding domain and impairing p53 mediated transcriptional transactivation (52). Heterogeneous or homogeneous *TP53* deletions that lead to decreased *TP53* gene dosage occur in ~ 10% of DLBCL cases (52, 55). BCL6, which is frequently dyregulated in DLBCL, binds to the *TP53* promoter region and suppresses the transcription of *TP53* gene (56). *MDM2* is amplified in a subset of DLBCL, contributing to increased ubiquitylation and degradation of p53 as well as decreased nuclear accumulation and transcriptional activity (57). MDM4, a protein that shares structural similarity with MDM2, inhibits p53 transcriptional activity. Amplifications of *MDM4* are also detected in DLBCL, possibly decreasing transcriptional activity of p53 (57). *ARF*, which encodes a protein that promotes MDM2 degradation and stabilizes p53, is frequently deleted in DLBCL, as a part of the *CDKN2A* locus (55, 58). *ARF* deletion contributes to the pathogenesis of DLBCL, at least partly via decreasing p53 protein level. Deletions of *KDM6B* and *RPL26*, which are located with *TP53* in the 17p13.1 region, are recurrent in DLBCL (57). Under normal conditions, the demethylase KDM6B can activate *ARF* transcription by demethylating the *p14ARF* locus (59). Additionally, KDM6B directly interacts with p53 by demethylating p53 and causes accumulation of p53 in the nucleus, thereby activating the function of p53 (60). Therefore, loss of *KDM6B* might decrease *ARF* transcription and p53 stability and p53 nuclear accumulation, thereby leading to p53 dysregulation.

RPL26 can bind the 5′ UTR of *TP53* and facilitate p53 translation, which increases stress-induced p53 expression (61). Thus, lack of RPL26 may decrease p53 expression by impeding p53 translation. Several other genetic aberrations may attenuate p53 protein function. ATM, which phosphorylates and activates p53, is recurrently mutated in DLBCL and may lead to impaired activation of p53 (16). Both CREBBP and EP300 can acetylate p53 and activate p53 transcriptional activity (62). Mutations in CREBBP and EP300 occur in DLBCL inactivating the acetyltransferase activity of these two proteins, thereby impairing p53 acetylation and activity (63).

### Tumor Microenvironment (TME) Dysfunction

**Figure 3:**
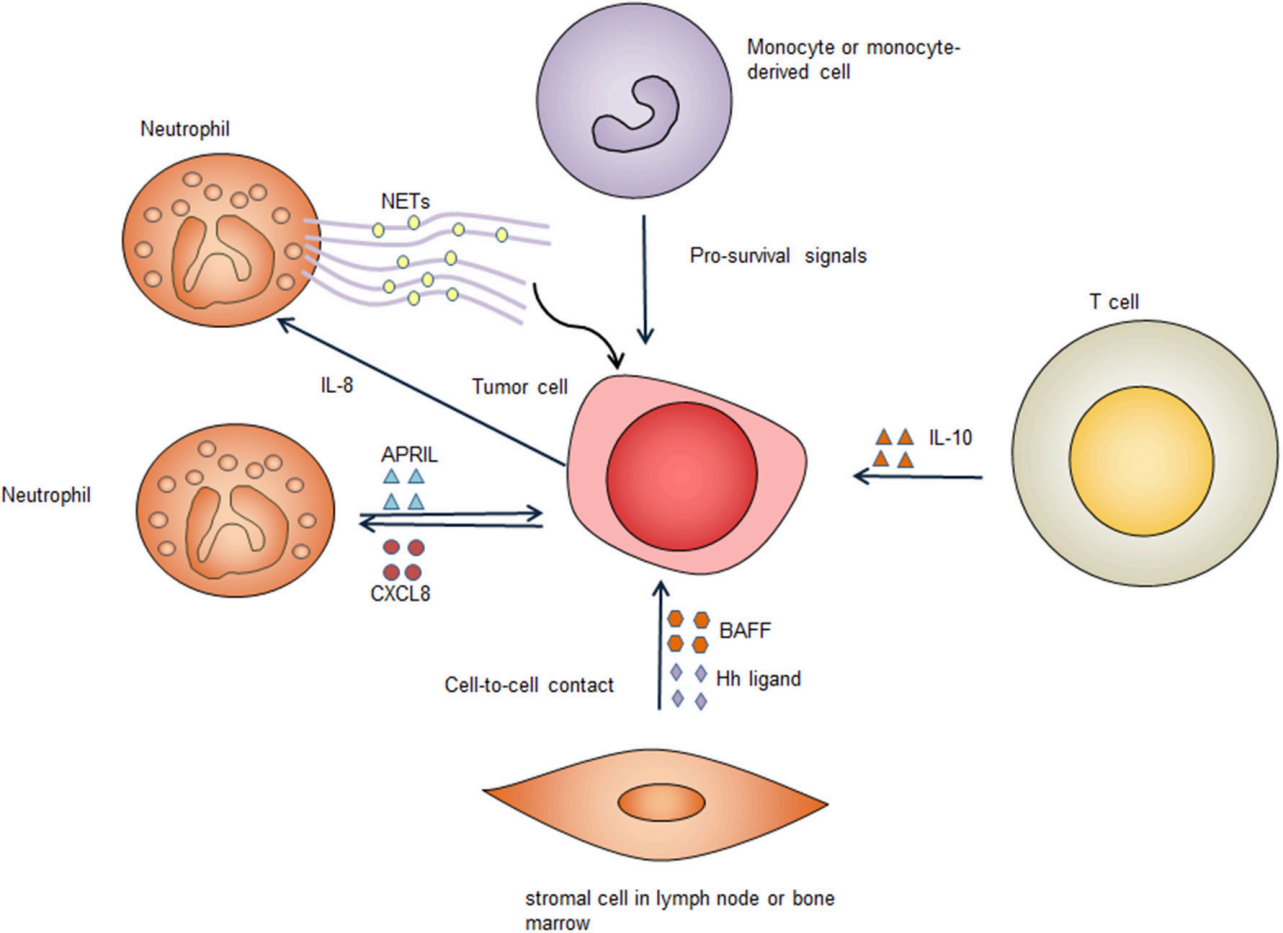
Survival signals from the tumor microenvironment. Immune cells and stromal cells promote tumor cell survival through cell-to-cell contact and secreting pro-survival factors. Several types of cells in the tumor microenvironment secret pro-survival factors including B-cell activating factor (BAFF), a proliferation-inducing ligand (APRIL), hedgehog (Hh) ligands, and interleukin-10 (IL-10). These factors bind to corresponding receptors on the tumor cell surface, providing important pro-survival signals to the tumor cells. Neutrophils form neutrophil extracellular traps (NETs), which activate Toll-like receptor 9 (TLR9) pathway to promote survival of diffuse large B cell tumor cells. CXCL8, C-X-C motif chemokine ligand 8; IL-8, interleukin-8.

The TME consists of vasculature, immune cells, fibroblasts, signaling molecules, and the extracellular matrix. In contrast with low-grade B cell lymphomas, DLBCL is relatively less dependent on the TME. Nevertheless, there is evidence suggesting that the TME provides indispensable survival signals in at least some cases of DLBCL. Not all DLBCL cells can be cultured *in vitro*, suggesting that these cells might be dependent on the TME to survive (64). Furthermore, in clinical settings the presence of certain types of immune cells predicts a poorer prognosis in DLBCL patients, suggesting that these immune cells might support the survival of DLBCL tumor cells or mediate lymphoma cell drug-resistance (65–68). Immune escape definitely contributes to the survival of DLBCL cells, however, this is not the focus of our review. Here, we discuss how the TME provides pro-survival signals to tumor cells. Several types of cells in the TME can provide survival signals for DLBCL cells (Figure 3).

#### Neutrophils

Coculture of neutrophils with DLBCL cell lines can sustain the survival of DLBCL cell lines (69). This effect is associated with decreased DLBCL cell apoptosis, suggesting that neutrophils promote the survival of DLBCL cells (69). In DLBCL, tumor-infiltrating neutrophils can secrete a proliferation-inducing ligand (APRIL) and cause overexpression of APRIL in DLBCL tissues, which could be related to poorer outcome of DLBCL patients (70). APRIL, which is a ligand of tumor necrosis factor (TNF) superfamily, can bind to B cell maturation antigen (BCMA) and the transmembrane activator and calcium modulator cyclophilin ligand interactor (TACI) and has a crucial role in regulating B cell survival (71). Mechanistically, DLBCL can produce C-X-C motif chemokine ligand 8 (CXCL8), which can recruit neutrophils expressing APRIL, thereby providing the tumor cells with pro-survival signals (72). Additionally, DLBCL derived interleukin-8 (IL-8) can induce neutrophils to form neutrophil extracellular traps (NETs) by binding to its receptor CXCR2 on neutrophils. The formed NETs directly activates TLR9 pathways in DLBCL, leading up-regulation of NF-κB, STAT3 and p38 pathways, which provide pro-survival signals (73).

#### Stromal Cells

Bone marrow stromal cells protect DLBCL cell lines and primary DLBCL cells through a combination of soluble factors and cell-to-cell contact. When co-cultured with mouse bone marrow stromal cells, primary human DLBCL cells have significantly increased clonogenicity and tumorigenicity (74). Bone marrow stromal cells secret B-cell activating factor (BAFF) and promote survival and drug-resistance of DLBCL cell lines. The adhesion of bone marrow stromal cells to DLBCL cells also can lead to activation of NF-κB pathway, which further up-regulates anti-apoptotic proteins c-IAPs and XIAP, leading to improved survival of lymphoma cells (75). These data suggest that bone marrow stromal cells have an important role in enhancing the survival of DLBCL tumor cells in the bone marrow microenvironment. Additionally, stromal cells can express hedgehog ligands and activate hedgehog signaling in B cell malignancies including DLBCL, promoting survival of these lymphoma cells (76). Co-culture of DLBCL cells with stromal cells results in up-regulation of BCL2, BCL-xL, and BCL2A1, which enhance cell survival (77). Inhibition of hedgehog signaling triggers apoptosis via down-regulating BCL2, suggesting the pro-survival effect provided by stromal cells is mediated, at least partly, by up-regulation of BCL2 (76). Stromal cells induced hedgehog signaling also contribute to chemoresistance by activating the transcription of adenosine triphosphate-binding cassette drug transporter ABCG2, thereby preventing DLBCL cells from cell death caused by chemotherapy (77). In addition to directly supporting the survival of DLBCL cells, stromal cells also recruits monocytes and neutrophils, which can provide additional pro-survival signals (73).

#### Other Types of Cells

Expansion of CD14 (+) CD169 (–) monocyte-derived cells that have dendritic cell differentiation potential is frequent in DLBCL (78). Coculture of monocytes with DLBCL cells can prolong the survival of the lymphoma cells (79). The mechanisms underlying monocyte support for DLBCL survival are less clear, and the effects are not mediated by BAFF, suggesting other factors may play a role (79). It is known that follicular helper T (Tfh) cells have a crucial role in the development of B cells. It remains to be determined if Tfh cells in the TME have a role in promoting the survival of DLBCL lymphoma cells. It has been shown that circulating CXCR5^+^CD4^+^ T cells enhance the survival of primary DLBCL cells through IL-10, indicating that these T cells may have a similar role in the TME (80).

These studies, in combination, support a role for the TME in enhancing DLBCL cell survival.

### Epstein-Barr Virus (EBV) Infection

**Figure 4:**
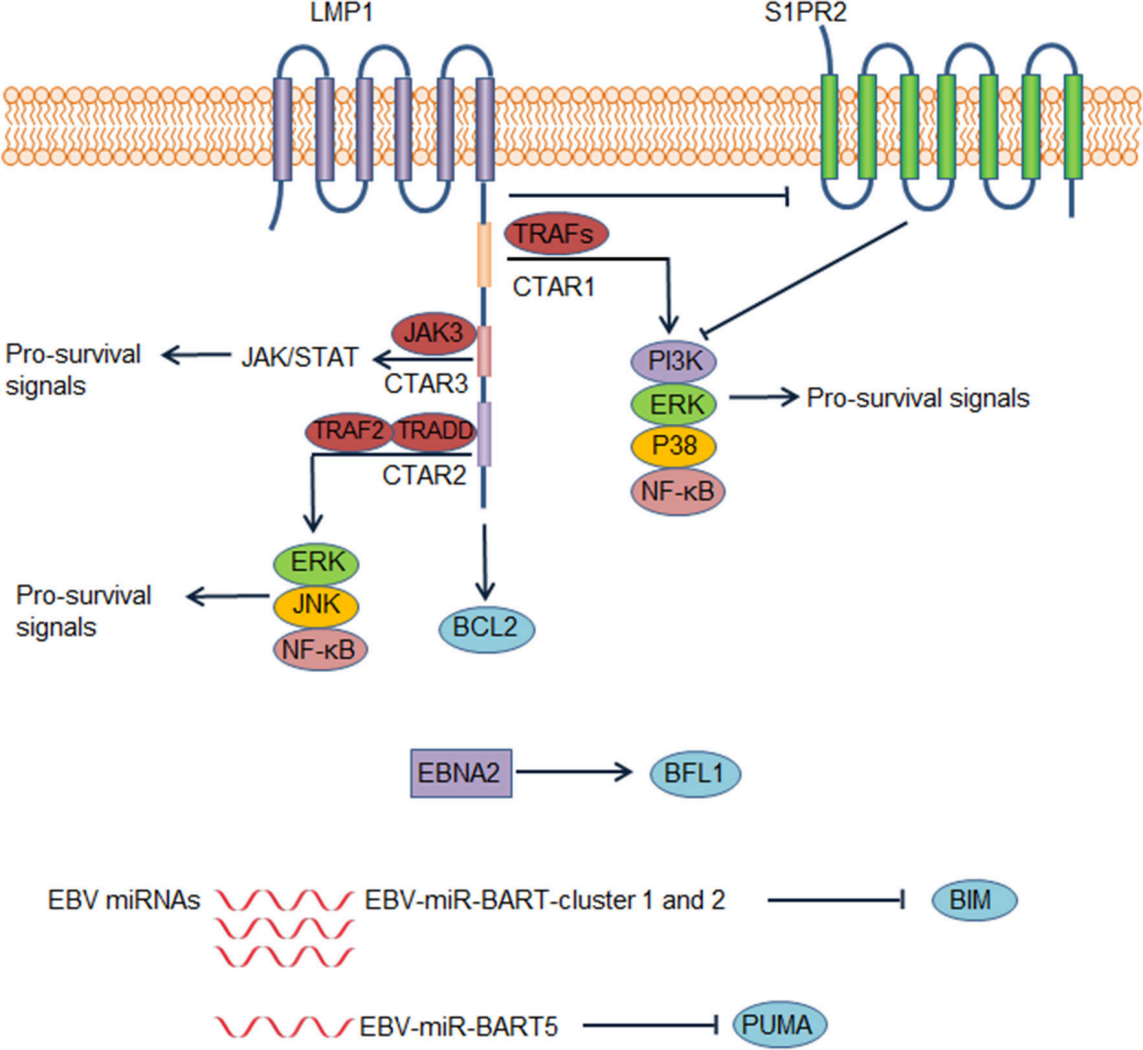
Epstein-Barr virus (EBV) contributes to dysregulation of survival of diffuse large B cell lymphoma (DLBCL) cells. In EBV positive DLBCL, LMP1, EBV nuclear antigen 2 (EBNA2), and several EBV microRNAs could lead to abnormal survival of DLBCL cells, contributing to the pathogenesis of EBV positive DLBCL. These factors lead to enhanced survival of DLBCL cells by increasing pro-survival signals or suppressing pro-apoptotic proteins. Abbreviations: BART, Bam HI A region rightward transcript; CTAR, C-terminal activation region; TRADD, tumor necrosis factor receptor type 1–associated death domain; TRAF, tumor necrosis factor receptor associated factor.

EBV infects B cells in most human hosts and is related to several types of B cell lymphoma. EBV-positive DLBCL, not otherwise specified, which accounts for ~ 10% of DLBCL (81), has been recognized as a distinct lymphoma entity. This disease usually occurs in individuals aged >50 years but can also occur in younger patients (82). Although elderly patients have an unfavorable outcome, the prognosis of young patients is excellent (82). By regulating different pathways, EBV promotes the survival of B cells and is implicated in DLBCL pathogenesis. EBV nuclear antigen 2 (EBNA2) and latent membrane protein 1 (LMP1) are expressed in 7–36% and over 90% of EBV^+^ DLBCL cases, respectively, suggesting type III and more frequently type II EBV latency (82). It is recognized that LMP1 is indispensable for prolonging the survival of transformed B cells (83). By mimicking the TNF-receptor superfamily member CD40, LMP1 activates the NF-κB pathway and several other pathways to promote cell proliferation and survival in transformed B cells (84, 85) (Figure 4). The cytoplasmic c-terminal tail of LMP1 has two effector domains, C-terminal activation regions 1 and 2 (CTAR1 and 2), which are essential for downstream NF-κB activation (85). CTAR1 recruits and interacts with TNF receptor associated factor (TRAF) members, thus activating downstream NF-κB, PI3K/AKT, p38 MAPK, and the ERK/MAPK pathway (86–88). CTAR2 recruits TNF receptor-associated death domain proteins to activate downstream pathways, including NF-κB, JNK, and the ERK/MAPK pathway (85, 87). Another effector domain, CTAR3, is located between CTAR1 and CTAR2 and can interact with JAK3 and activate the JAK/STAT pathway (89). Additionally, LMP1 induces BCL2 expression in infected B cells and prevents these cells from undergoing apoptosis (90). Accordingly, BCL2 is virtually always positive in EBV^+^ DLBCL (91). LMP1 down-regulates the expression of a sphingosine-1-phosphate (S1P) receptor S1PR2 at the transcriptional level, a molecule that inhibits PI3K/AKT pathway, contributing to PI3K/AKT pathway activation (92). EBNA2 also up-regulates the anti-apoptotic protein BFL1 (93). Some EBV miRNAs improve the survival of B cells by targeting pro-apoptotic proteins. For example, EBV-miR-Bam HI A region rightward transcript (BART)-cluster 1 and 2 and EBV-miR-BART5 target the pro-apoptotic proteins BIM and PUMA, respectively, thereby increasing the survival of infected B cells (94, 95).

## Therapeutic Strategies

An increasing number of novel drugs targeting aberrant cell survival through modulating different pathways have shown efficacy in preclinical studies and/or clinical trials, with some of these drugs showing moderate or significant activities (1, 96). These drugs targeting different pathways are summarized in Table 1. Ongoing trials testing some of these drugs in DLBCL have been summarized in Table 2.

**Table 1:** Selected drugs targeting aberrant cell survival in diffuse B cell lymphoma.

**Pathways**	**Targets**	**Drugs**
BCR signaling	BTK	Ibrutinib, BGB-3111, acalabrutinib, TG-1701
	MALT1	Phenothiazines, MI-2
	PKC-β	Enzastaurin
	SYK	Fostamatinib, entospletinib, TAK-659
	PI3K	Copanlisib, buparlisib, CUDC-907, idelalisib, YY-20394, INCB050465, TGR-1202
	AKT	AZD5363, MK2206
	mTOR	Everolimus, temsirolimus
BCL2 family	BCL2	Navitoclax, venetoclax, obatoclax
	MCL1	S63845, AMG176
The p53 pathway	p53	APR-246
	MDM2	Nutlin-3a, idasanutlin
	MDM4	XI-011
	XPO1	Selinexor

*BTK, Bruton tyrosine kinase; PKCβ, Protein kinase C β; SYK, spleen tyrosine kinase*.

**Table 2:** Ongoing trials with drugs targeting cell survival in diffuse large B cell lymphoma.

**Target**	**Drug**	**NCT number**	**Phase**	**Indication^a^**	**Treatments^b^**
BTK	Ibrutinib	NCT02733042	I/II	R/R lymphoma or CLL	Durvalumab + lenalidomide ± R; Durvalumab+ibrutinib; Durvalumab + bendamustine ± R; Durvalumab monotherapy
BTK	Ibrutinib	NCT01479842	I	R/R DLBCL, MCL, or indolent NHL	R + bendamustine + ibrutinib
BTK	Ibrutinib	NCT02436707	II	R/R aggressive B-cell lymphoma	R-GDP; Ibrutinib+R-GDP; R + cisplatin + mesna + cyclophosphamide + etoposide + G-CSF
BTK	Ibrutinib	NCT02950220	I	R/R NHL	Ibrutinib + pembrolizumab
BTK	Ibrutinib	NCT02747732	II	R/R aggressive B cell lymphoma	Ibrutinib + bendamustine + R
BTK	Ibrutinib	NCT01109069	II	R/R B cell Lymphoma	Ibrutinib
BTK	Ibrutinib	NCT03479268	I	R/R CLL or NHL	Ibrutinib + pevonedistat
BTK	Ibrutinib	NCT01955499	I	R/R B-cell NHL	Ibrutinib + lenalidomide
BTK	Ibrutinib	NCT02955628	II	Transplant-eligible R/R DLBCL	Ibrutinib + RICE
BTK	Ibrutinib	NCT03770416	II	R/R central nervous system lymphoma	Ibrutinib + nivolumab
BTK	Ibrutinib	NCT02401048	I/II	R/R DLBCL or FL	Ibrutinib + MEDI4736
BTK	Ibrutinib	NCT02077166	I/II	R DLBCL	Ibrutinib + lenalidomide + R
BTK	Ibrutinib	NCT02203526	I	PCNSL	Ibrutinib followed by DA-TEDDI-R
BTK	Ibrutinib	NCT02692248	II	R/R non-GCB DLBCL (non-candidates for ASCT)	Ibrutinib + R + gemcitabine + oxaliplatin + dexamethasone followed by ibrutinib
BTK	Ibrutinib	NCT02315326	I/II	R/R PCNSL and SCNSL	Ibrutinib; ibrutinib + HD-MTX; ibrutinib + R + HD-MTX
BTK	Ibrutinib	NCT03703167	I	R/R PCNSL and SCNSL	Ibrutinib + R + lenalidomide
BTK	Ibrutinib	NCT02760485	I/II	R/R DLBCL	Ibrutinib + itacitinib
BTK	Ibrutinib	NCT02703272	III	Pediatric and young adult patients with R/R mature B-cell NHL	Ibrutinib + RICE/RVICI; RICE/RVICI
BTK	Ibrutinib	NCT03684694	I	R/R DLBCL or MCL	Ibrutinib + loncastuximab tesirine
BTK	Ibrutinib	NCT02542514	II	R/R PCNSL or intraocular Lymphoma	Ibrutinib
BTK	Ibrutinib	NCT03220022	I	HIV-positive stage II-IV DLBCL	Ibrutinib + R-DA-EPOCH
BTK	Ibrutinib	NCT03129828	I/II	Elderly patients With CD20^+^ DLBCL, IPI ≥ 2	Ibrutinib + bortezomib + R-CHOP
BTK	Ibrutinib	NCT01855750	III	Newly diagnosed non-GCB DLBCL	Ibrutinib + R-CHOP vs. placebo + R-CHOP
BTK	Ibrutinib	NCT02670616	II	Newly diagnosed Epstein-Barr virus-positive DLBCL	Ibrutinib + R-CHOP
BTK	Ibrutinib	NCT03399513	II	Untreated younger, high-risk patients with DLBCL	Ibrutinib and R-CHOEP-14
BTK	Ibrutinib	NCT02636322	II	Untreated high risk DLBCL	Lenalidomide + ibrutinib + R-DA-EPOCH; Lenalidomide + ibrutinib + R-CHOP
BTK	Ibrutinib	NCT02623010	II	Newly diagnosed PCNSL	Ibrutinib maintenance
BTK	BGB-3111	NCT02795182	I	R/R B cell malignancies	BGB-3111 + BGB-A317
BTK	BGB-3111	NCT03145064	II	R/R non-GCB DLBCL	BGB-3111
BTK	BGB-3111	NCT02343120	I	R/R B cell lymphoma	BGB-3111
BTK	BGB-3111	NCT03189524	I	R/R B cell lymphoma	BGB-3111
BTK	Acalabrutinib	NCT02362035	Ib/II	R/R hematologic malignancies	Acalabrutinib + pembrolizumab
BTK	Acalabrutinib	NCT03736616	II	R/R DLBCL	Acalabrutinib + RICE
BTK	Acalabrutinib	NCT03571308	Ib/II	Untreated DLBCL	Acalabrutinib + R-CHOP
PKC-β	Enzastaurin	NCT03263026	III	Untreated DLBCL with genomic biomarker DGM1™	Enzastaurin + R-CHOP vs. R-CHOP
SYK	entospletinib	NCT03225924	I/II	Newly diagnosed DLBCL (aaIPI ≥ 1)	Entospletinib+R-CHOP vs. R-CHOP
SYK	TAK-659	NCT02954406	I	R/R advanced NHL	TAK-659 + bendamustine; TAK-659 + bendamustine + R; TAK-659 + gemcitabine; TAK-659 + lenalidomide; TAK-659 + ibrutinib
SYK	TAK-659	NCT02000934	I	Advanced solid tumor and R/R lymphoma	TAK-659
SYK	TAK-659	NCT03238651	I	R/R NHL	TAK-659
SYK	TAK-659	NCT03123393	II	R/R DLBCL	TAK-659
SYK	TAK-659	NCT03772288	I	R/R advanced NHL	TAK-659 + NKTR-214
SYK	TAK-659	NCT03742258	I	Newly diagnosed high-risk DLBCL	TAK-659 + R-CHOP
PI3K	Copanlisib	NCT01660451	II	R/R indolent or aggressive NHL	Copanlisib
PI3K	Copanlisib	NCT03484819	II	R/R DLBCL and PMBCL	Copanlisib + nivolumab
PI3K	Copanlisib	NCT03502733	I	Metastatic solid tumors or lymphoma	Copanlisib + nivolumab
PI3K	CUDC-907	NCT02909777	I	R/R solid tumors, CNS tumors, or lymphoma	CUDC-907
PI3K	CUDC-907	NCT02674750	II	R/R DLBCL	CUDC-907
PI3K	Idelalisib	NCT03151057	I	Post allogeneic HSCT in B cell malignancies	Idelalisib
PI3K	Idelalisib	NCT03576443	II	R/R DLBCL	Idelalisib
PI3K	TGR-1202	NCT01767766	I	R/R hematological malignancies	TGR-1202
PI3K	TGR-1202	NCT02006485	I	R/R B cell malignancies	TGR-1202 + ublituximab; TGR-1202 + ublituximab + ibrutinib; TGR-1202 + ublituximab + bendamustine
PI3K	TGR-1202	NCT02793583	IIb	R/R NHL	TGR-1202 + ublituximab; TGR-1202; TGR-1202 + ublituximab + bendamustine
PI3K	TGR-1202	NCT03283137	I	R/R CLL and B cell NHL	TGR-1202+ pembrolizumab
PI3K	TGR-1202	NCT02867618	I/II	R/R lymphoma	TGR-1202+ carfilzomib
PI3K	YY-20394	NCT03757000	I	R/R B cell malignancies	YY-20394
PI3K	INCB050465	NCT03424122	I	R/R B cell lymphoma	INCB050465 + R; INCB050465 + bendamustine + R; INCB050465 + ibrutinib
mTOR	Everolimus	NCT01075321	I/II	R/R NHL	Everolimus + lenalidomide
mTOR	Everolimus	NCT00918333	I/II	R/R MM, NHL or HL	Everolimus + panobinostat
mTOR	Everolimus	NCT01665768	II	After high-dose consolidative therapy in lymphoma	Maintenance: everolimus + R
mTOR	Everolimus	NCT00474929	I/II	R/R lymphoma or MM	Everolimus + sorafenib
mTOR	Everolimus	NCT00436618	II	R/R lymphoma	Everolimus
mTOR	Temsirolimus	NCT01076543	I/II	R/R HL or NHL	Temsirolimus and lenalidomide
BCL2	Venetoclax	NCT03236857	I	Pediatric and young adult patients With R/R malignancies	Venetoclax ± chemotherapy
BCL2	Venetoclax	NCT02265731	I/II	R/R hematologic malignancies	Venetoclax
BCL2	Venetoclax	NCT03797261	I	R/R hematologic malignancies	Venetoclax + AMG 176
BCL2	Venetoclax	NCT03583424	I/II	R/R NHL	Venetoclax + BEAM conditioning for ASCT
BCL2	Venetoclax	NCT02992522	I	R/R B-cell NHL	Venetoclax + obinutuzumab + lenalidomide
BCL2	Venetoclax	NCT03082209	I	R/R solid tumors and hematologic malignancies	Venetoclax + ABBV-621
BCL2	Venetoclax	NCT02391480	I	R/R cancer	ABBV-075; venetoclax + ABBV-075
BCL2	Venetoclax	NCT01328626	I	R/R CLL or NHL	Venetoclax
BCL2	Venetoclax	NCT02987400	II	R/R DLBCL	Venetoclax + obinutuzumab
BCL2	Venetoclax	NCT02611323	I	R/R FL or DLBCL	Venetoclax + R + polatuzumab vedotin
BCL2	Venetoclax	NCT01594229	I	R/R NHL	Venetoclax + bendamustine + R
BCL2	Venetoclax	NCT03276468	I	R/R lymphoma	Venetoclax + atezolizumab + obinutuzumab
BCL2	Venetoclax	NCT03255096	I	R/R DLBCL and/or HGBL and/or HGBL with MYC and/or BCL2 and/or BCL6 gene rearrangements	Venetoclax + RO6870810 ± R
BCL2	Venetoclax	NCT03064867	I	R/R DLBCL	Venetoclax plus R-ICE
BCL2	Venetoclax	NCT03713580	I	R/R NHL (eligible for ASCT)	Venetoclax + BEAM conditioning regimen
BCL2	Venetoclax	NCT03036904	I	Untreated aggressive B-cell lymphoma	Venetoclax+ R-DA-EPOCH
BCL2	Venetoclax	NCT02055820	Ib/II	Untreated DLBCL	Venetoclax + CHOP + obinutuzumab or rituximab
MDM2	Idasanutlin	NCT02624986	Ib/II	R/R DLBCL or FL	Idasanutlin + obinutuzumab or R
XPO1	Selinexor	NCT02323880	I	Younger R/R solid tumors or high-grade gliomas	Selinexor
XPO1	Selinexor	NCT02471911	I	R/R aggressive B cell lymphoma	Selinexor + RICE
XPO1	Selinexor	NCT02741388	I	R/R B cell lymphoma	Selinexor + R-DHAOx; selinexor + R-GDP
XPO1	Selinexor	NCT03147885	Ib/II	Newly diagnosed B cell NHL	Selinexor + R-CHOP
BTK + BCL2	Ibrutinib + venetoclax	NCT03223610	I	R/R B cell lymphoma	Ibrutinib + venetoclax + prednisone + obinutuzumab + lenalidomide
BTK + BCL2	Ibrutinib + venetoclax	NCT03136497	I	R/R DLBCL	Ibrutinib + venetoclax + R
BTK+PI3K	TG-1701 + TGR-1202	NCT03671590	I	R/R NHL or CLL	TG-1701 + TGR-1202 + ublituximab
BTK+PI3K	Ibrutinib + buparlisib	NCT02756247	I	R/R MCL, FL and DLBCL	Ibrutinib + buparlisib
BTK+PI3K	Ibrutinib + TGR-1202	NCT02874404	II	R/R DLBCL	Ibrutinib + TGR-1202
BTK+PI3K	Ibrutinib + copanlisib	NCT03581942	IB/II	R/R PCNSL	Ibrutinib + copanlisib
SYK+BCL2	TAK-659 + venetoclax	NCT03357627	I	R/R NHL	TAK-659 + venetoclax
BTK+XPO1	Ibrutinib + selinexor	NCT02303392	I	R/R CLL or aggressive NHL	Ibrutinib + selinexor
BCL2 + MDM2	Venetoclax + idasanutlin	NCT03135262	Ib/II	R/R DLBCL or FL	Venetoclax + idasanutlin + obinutuzumab or R

a *These ongoing clinical trials are for diffuse large B cell lymphoma or include diffuse large B cell lymphoma as well as other types of tumors*;

b *This column described treatments for diffuse large B cell lymphoma*.

*aaIPI, age-adjusted international prognostic index; ABC, activated B-cell; ASCT, autologous stem cell transplant; BEAM, carmustine, etoposide, cytarabine and melphalan; BTK, Bruton tyrosine kinase; CHOP, cyclophosphamide, doxorubicin, vincristine, and prednisone; CLL, chronic lymphocytic leukemia; CNS, central nervous system; DA-TEDDI-R, temozolomide, etoposide, liposomal doxorubicin, dexamethasone, ibrutinib and rituximab; DLBCL, diffuse large B cell lymphoma; FL, follicular lymphoma; GCB, germinal center B-cell; G-CSF, granulocyte-colony stimulating factor; HD-MTX, high-dose methotrexate; HGBL, high-grade B cell lymphoma; HL, Hodgkin lymphoma; HSCT, hematopoietic stem cell transplantation; IPI, international prognostic index; MCL, mantle cell lymphoma; MM, multiple myeloma; NHL, non-Hodgkin lymphoma; PCNSL, primary central nervous system lymphoma; PMBCL, primary mediastinal large B-Cell lymphoma; R, rituximab; R-CHOP, rituximab plus cyclophosphamide, doxorubicin, vincristine, and prednisone; R-CHOEP-14, rituximab plus cyclophosphamide, doxorubicin, vincristine, etoposide, and prednisone every 2 weeks; R-DA-EPOCH, rituximab with dose-adjusted etoposide, prednisone, vincristine, cyclophosphamide, and doxorubicin; R-DHAOx, rituximab, dexamethasone, high-dose cytarabine, and oxaliplatin; R-GDP, rituximab, gemcitabine, cisplatin, and dexamethasone; RICE, rituximab, ifosfamide, carboplatin, and etoposide; R/R, relapsed or refractory; RVICI, rituximab, vincristine, ifosfamide, carboplatin, and idarubicin; SCNSL, secondary central nervous system lymphoma*.

### Targeting the BCR Pathway

The BCR pathway has emerged as a crucial target for treating DLBCL patients. Several inhibitors that target molecules in the BCR signaling pathway have shown efficacy in preclinical studies and/or clinical trials. BTK is an important target for suppressing BCR signaling and inhibiting BTK impairs the survival of ABC DLBCL cell lines. The BTK inhibitor ibrutinib has shown significant efficacy in relapsed/refractory ABC-DLBCL patients, especially in those tumors with concurrent *MYD88*^L265P^ and *CD79B* mutations (97). The combination of ibrutinib with immunochemotherapy has achieved promising responses in newly-diagnosed DLBCL or refractory/relapsed DLBCL patients (98, 99). Other BTK inhibitors, including BGB-3111 and acalabrutinib, are now being tested in DLBCL. MALT1, which acts as an adaptor protein to activate the downstream NF-kB pathway, has emerged as a potential therapeutic target (100). Several small molecule MALT1 inhibitors, which inhibit the protease activity of MALT1, have shown remarkable anti-tumor effects on ABC DLBCL cells *in vitro* and *in vivo* (101, 102). As a key signaling hub downstream of BCR signaling, PKC-β has been an attractive target for treating DLBCL and the PKC-β inhibitor enzastaurin has been tested in refractory/relapsed or newly-diagnosed DLBCL patients. For refractory/relapsed DLBCL, only a small proportion of patients responded to single agent enzastaurin (103). Additionally, incorporating enzastaurin into induction chemotherapy in newly diagnosed DLBCL patients or using enzastaurin as maintenance after CR in high-risk DLBCL did not provide significant survival benefits (104, 105). SYK dependent tonic BCR signaling is indispensable for the survival of DLBCL cell lines, which provides a rationale for targeting SYK in the treatment of DLBCL (20). The selective SYK inhibitor fostamatinib has shown significant activity in refractory/relapsed DLBCL, with 5 of 23 patients achieving a response in a phase 1/2 study (106). Nonetheless, a phase 2 study including 68 refractory/relapsed DLBCL patients showed that only 3% of patients achieved a response (107).

The PI3K/AKT pathway is involved in the downstream of both chronic and tonic BCR signaling. Therefore, inhibiting PI3K/AKT pathway can be used as a strategy to suppress cell survival in both ABC DLBCL and GCB DLBCL. In ABC-DBCL cell lines with *CD79B* mutations, PI3K inhibition decreases NF-κB activity and impairs the survival of affected DLBCL cell lines (108). Additionally, in GCB DLBCL cell lines, pharmacologic PI3K inhibition selectively impairs survival of PTEN-deficient cell lines (109). A pan-class I PI3k inhibitor, copanlisib, has been tested in refractory/relapsed DLBCL patients as a single agent in phase 1 and 2 clinical trials (110, 111). In the phase 2 trial, the overall response rate (ORR) was 25% (10 of 40) with 5 CRs. The ORR was 13.6% with 1 CR in GCB-type patients and 37.5% with 4 CRs (25%) in ABC-type patients, suggesting that ABC-type had a better response to copanlisib (111). In an expanded phase 1 trial of CUDC-907, a dual inhibitor of histone deacetylase (HDAC) and PI3K, 11 of 30 evaluable patients had an objective response including 5 CR (112). AKT inhibitors showed activities in DLBCL in preclinical studies as well (113–115). The AKT inhibitor AZD5363 was effective in PTEN-deficient DLBCL irrespective of cell-of-origin subtype (115). Another AKT inhibitor, MK2206, also showed significant activity in preclinical DLBCL models, however, in a phase 2 trial, none of 9 evaluable patients treated with MK2206 had a response (116). Activation of PI3K/AKT contributes to activation of mTOR signaling, which promotes the survival of DLBCLs. In addition to PI3K/AKT activation, mTOR could also be driven by activated CARD11 or the My-T-BCR supercomplex (28). Inhibition of mTOR also could be a strategy to suppress the survival of DLBCL cells. The mTOR inhibitors everolimus and temsirolimus have shown significant single-agent activity in refractory/relapsed DLBCL patients, providing additional salvage therapeutic options (117, 118). Given the pathogenic role of the My-T-BCR supercomplex, combination of mTOR inhibitors with BCR inhibitors could be used in DLBCL depending on the My-T-BCR supercomplex (28), and BTK inhibitor ibrutinib and mTOR inhibitor are synergistic in suppressing the growth the xenografts of TMD8, which is dependent on the My-T-BCR supercomplex, supporting combinational use of BTK inhibitors and mTOR inhibitors in My-T-BCR supercomplex positive DLBCLs (28).

### Inhibiting the BCL2 Family Members

The role of BCL2 in DLBCL biology makes it a compelling target for treating DLBCL. Navitoclax (ABT-263), which inhibits both BCL2 and BCL-xL, has shown promising efficacy in B cell malignancies. Nonetheless, the thrombocytopenia caused by inhibiting BCL-xL limits the use of navitoclax. Venetoclax (ABT-199), which is a selective BCL2 inhibitor, has potent antitumor activity but spares platelets. In a phase 1 study which included 34 cases of refractory/relapsed DLBCL, 6 patients achieved objective response with 4 patients achieving CR (119). There are some clinical trials currently exploring the efficacy of combining venetoclax with other regimens or drugs in refractory/relapsed or newly-diagnosed DLBCL. MCL1 also can be targeted in DLBCL. Knockdown of MCL1 induces cell apoptosis in MCL1-positive DLBCL cell lines (43). Further, BCL2 inhibition in DLBCL cell lines results in up-regulation of MCL1 expression, possibly interfering with the therapeutic effect of venetoclax (120). Venetoclax and drugs targeting MCL1 displayed a synergistic effect in inducing apoptosis in preclinical models of high-risk DLBCL (120). A highly potent and selective inhibitor of MCL1, S63845 (MIK665), has shown significant anti-tumor activity in several DLBCL cell lines and Eμ-Myc lymphoma cells transplanted mouse models (121). One clinical trial (NCT02992483) is currently investigating the preliminary activity of S63845 in refractory/relapsed multiple myeloma or lymphoma including MYC positive DLBCL. Additionally, the pan-BCL2 inhibitor, obatoclax, which inhibits BCL2, BCL-xL, and MCL1, induces apoptotic cell death in DLBCL cell lines including MCL1 positive ones (43, 122).

### Targeting Aberrant p53 Pathway

Targeting an aberrant p53 pathway could be used potentially to restore the function of p53 and trigger cell death. One low-molecular-weight compound, APR-246, is capable of restoring the transcriptional activation function of mutant p53, thus inducing apoptosis in human cancer cells (123). The safety and activity of APR-246 has been tested in refractory/relapsed hematological malignancies including 3 non-Hodgkin lymphoma cases in a phase 1 clinical trial (124). In some patients, tumor cells exhibited upregulation of p53 targeted genes and increase apoptosis. Nonetheless, the efficacy of APR-246 in DLBCL remains to be determined. Disrupting the p53-MDM2 interaction impairs MDM2-mediated p53 degradation, thereby increasing p53 stability and expression. Nutlin-3a disrupts the p53-MDM2 interaction and activates p53, thereby up-regulating the pro-apoptotic proteins BAX and PUMA and inducing apoptosis in DLBCL cell lines with t(14;18)(q32;q21) (125). Idasanutlin, a potent and selective MDM2 antagonist, when combined with obinutuzumab and venetoclax, showed significant antitumor activity in a xenograft model of DLBCL (126). This three-drug combination remarkably improved the tumor-free survival of mice. Idasanutlin in combination with rituximab and venetoclax is currently investigated in refractory/relapsed DLBCL patients (NCT03135262). One MDM4 inhibitor, XI-011, induced apoptosis in breast cancer cells by activating p53 (127). The efficacy of MDM4 inhibitors remains to be determined in DLBCL cells. The transcriptional activation activity of p53 depends on its nuclear localization. XPO1 mediates the nuclear export of proteins including p53. Selinexor, an inhibitor of XPO1, inhibits the nuclear export of p53 and restores p53 nuclear localization (128). In a phase 1 trial, 13 of 41 DLBCL patients treated with single agent selinexor had an objective response, with 4 CR (128).

### Strategies for Using Novel Drugs to Target Cell Death Pathway in DLBCL

Combination of novel drugs can improve therapeutic effects thorough additive effects or synergistic effects. By using a high-throughput platform to test compounds, Mathews et al. found that ibrutinib was able to cooperate with other drugs including inhibitors of PI3K pathway (agents targeting the PI3K catalytic subunit, AKT, and the mTORC1 complex), BCL2 inhibitors, and cytotoxic chemotherapeutic drugs in killing ABC DLBCL cells (129). This provides a rationale for use of combination of ibrutinib and other drugs. Furthermore, ibrutinib-resistant TMD8 cells (ABC DLBCL) had elevated BCL2 expression and were vulnerable to BCL2 inhibition; samples from ABC DLBCL patients who had poorer responses to ibrutinib also exhibited higher BCL2 expression (130). These findings support the combination of ibrutinib and BCL2 inhibitors in the treatment of ABC DLBCL (130).

The prior treatments, age and performance status need to be taken into consideration when using novel drugs in the treatment of relapsed/refractory DLBCL. It needs to be pointed out that, the ORR is low in relapsed/refractory DLBCL treated with novel drug monotherapies (97, 119). Even for patients who have achieved responses, the responses are not durable. In relapsed/refractory DLBCL patients receiving single-agent ibrutinib, the median response duration was only 4.83 months in ABC DLBCL, where ibrutinib shows significantly better activity, although patients achieving CR had a relatively long remission (97). Several strategies could possibly be used to apply novel agents in the treatment of DLBCL. In relapsed/refractory DLBCL, combining novel drugs or combining novel drugs with conventional salvage immunochemotherapies could be used to produce higher response rates; for instance, in a phase 1 trial, in 20 patients receiving ibrutinib in combination with R-ICE, 18 patients had a response, with 11 achieving CR (99). In this setting, autologous stem cell transplantation could be used as consolidation therapy in responding patients who are eligible for autologous stem cell transplantation. In the phase 1 trial of venetoclax in relapsed/refractory DLBCL, one DLBCL patient, who proceeded to allogeneic stem cell transplantation (SCT) after achieving CR with venetoclax, achieved durable remission (119). This suggests allogeneic SCT could possibly be used to as a consolidative treatment for patients who have achieved remission with novel drugs. More importantly, the chimeric antigen receptor (CAR) T-cell therapy produced significantly higher CR rates and durable remissions in relapsed/refractory DLBCL (131, 132). Therefore, these novel drugs could be used as bridging therapies to the CAR T-cell therapy or in combination with the CAR T-cell therapy. Additional efforts have been made to improve the outcome of DLBCL patients by incorporating novel drugs into R-CHOP induction in the first-line setting (Table 2). A recent study showed that the addition of ibrutinib to R-CHOP significantly improved the outcome of non-GCB DLBCL patients younger than 65 years (133).

## Conclusion

Cell survival dysregulation represents a hallmark of DLBCL. Disruptions of multiple cellular pathways, including BCR signaling, the BCL2 pathway, the p53 pathway and so on, contribute to dysregulated cell survival in DLBCL. Tumor microenvironment dysfunction as well as EBV infection are also involved in promoting the survival of DLBCL cells. Several novel drugs that are capable of targeting abnormal cell survival pathways have been developed and show promising efficacy in DLBCL preclinical models and patients trials. However, until now, of the drugs discussed above, ibrutinib is the only one that has shown clinical benefit in both relapsed/refractory and untreated DLBCL patients (ABC/non-GCB subtype) (97, 133). The success of ibrutinib suggests that ABC cases, especially those with My-T-BCR supercomplex expression, are highly dependent on the BCR signaling (28). This also highlights the importance of BTK molecule in the biology of these DLBCL cases. The failure of some drugs in clinical trials could be attributed to, but not to restricted to the following reasons. DLBCL is a heterogeneous disease (16), different cases may depend on different pathways for cell survival; therefore, when a drug is tested in a group of DLBCL cases, only some of them achieve a response, highlighting the importance of identifying reliable predictors of response. Some cases can rapidly develop resistance mechanisms to a novel drug. Understanding these resistance mechanisms is helpful for developing novel strategies in the treatment of DLBCL. Further understanding of cell survival dysregulation in DLBCL will help to identify more potential targets to provide novel therapeutic options for DLBCL patients in the future.

## Author Contributions

YM conceptualized and wrote the manuscript and created the figures. ZX-M and KHY contributed to the conception and writing. ZX-M and LM revised the manuscript. JL contributed to the conception and critically revised the manuscript. All authors have read and approved the final manuscript.

### Conflict of Interest Statement

KHY receives research support from Roche Molecular System, Gilead Sciences Pharmaceutical, Seattle Genetics, Dai Sanyo Pharmaceutical, Adaptive Biotechnology, Incyte Pharmaceutical, and HTG Molecular Diagnostics. The funders played no role in the study design, the collection, analysis or interpretation of data, the writing of this paper or the decision to submit it for publication. The remaining authors declare that the research was conducted in the absence of any commercial or financial relationships that could be construed as a potential conflict of interest.
